# Microbiologically Confirmed Concomitant Tuberculous and Pneumococcal Meningitis in an Adult: A Case Report

**DOI:** 10.7759/cureus.114665

**Published:** 2026-08-17

**Authors:** Fatima Silvana, Amreesh Paul, Rakesh Kumar, Abhishek Mallik, Ritvik R Singh

**Affiliations:** 1 General Physician, Astromedics Hospital, Gorakhpur, IND; 2 Anesthesiology, Astromedics Hospital, Gorakhpur, IND; 3 Neurosurgery, Astromedics Hospital, Gorakhpur, IND; 4 General Surgery, Astromedics Hospital, Gorakhpur, IND; 5 General Medicine, Astromedics Hospital, Gorakhpur, IND

**Keywords:** anti tubercular therapy (att), meningitis, mri brain, pneumococcal meningitis, tubercular meningitis

## Abstract

Concurrent infection of the central nervous system by *Mycobacterium tuberculosis* and *Streptococcus pneumoniae *is rare and poses substantial diagnostic and therapeutic challenges due to the overlap of clinical symptoms and cerebrospinal fluid (CSF) findings. A 45-year-old male presented with complaints of fever, headache, vomiting, generalized seizures, and altered sensorium. Magnetic resonance imaging of the brain was normal, and CSF analysis showed lymphocytic pleocytosis, protein elevation, and elevated levels of adenosine deaminase. A Cartridge-Based Nucleic Acid Amplification Test performed on the CSF detected *Mycobacterium tuberculosis*. CSF culture grew *Streptococcus pneumoniae*, confirming the diagnosis of both tuberculous and pneumococcal meningitis in the patient. The treatment included pathogen-directed intravenous antibiotics, tuberculosis treatment, corticosteroids, and comprehensive neurocritical care, including mechanical ventilation. The patient also suffered from ventilator-associated pneumonia due to Klebsiella pneumoniae, which was managed successfully. The patient made steady neurological improvement, was weaned off mechanical ventilation, and was discharged. This case illustrates that diagnosing combined tuberculous and bacterial meningitis remains challenging, and laboratory confirmation of a single infection does not exclude additional pathogens or etiologies, especially in countries with prevalent tuberculosis. Comprehensive CSF evaluation using both molecular diagnostics and conventional culture enabled timely diagnosis and pathogen-directed therapy. This case highlights the importance of maintaining a high index of suspicion for dual central nervous system infections and integrating molecular diagnostics with conventional microbiological techniques to facilitate early pathogen-directed therapy.

## Introduction

Acute meningitis is a neurological emergency associated with substantial morbidity and mortality, despite advances in antimicrobial therapy and critical care. Streptococcus pneumoniae is the most common cause of community-acquired bacterial meningitis in adults and remains associated with a high risk of neurological disability and mortality despite appropriate antimicrobial therapy. Therefore, prompt diagnosis, early empirical antimicrobial therapy, and subsequent pathogen-directed treatment based on microbiological confirmation are essential for improving clinical outcomes [1,2].

Tuberculous meningitis (TBM), caused by *Mycobacterium tuberculosis*, is associated with a high mortality rate and neurological complications. TBM is different from acute pyogenic meningitis since it occurs in a more subacute form, characterized by vague symptoms that delay the diagnosis and treatment of the disease. Conventional cerebrospinal fluid (CSF) smear microscopy has limited sensitivity because TBM is paucibacillary, while the introduction of nucleic acid amplification techniques, including Cartridge-Based Nucleic Acid Amplification Test (CB-NAAT), has increased the speed and accuracy of the TBM diagnosis. The coexistence of pneumococcal and tuberculous meningitis is particularly unusual because these infections differ markedly in their tempo, pathophysiology, and diagnostic approach, and the clinical and CSF features of one may obscure the presence of the other. Recognizing such dual infection is clinically consequential, as delayed identification of either pathogen can lead to inadequate or incomplete treatment, with potentially devastating neurological outcomes [3,4].

Bacterial meningitis and TBM are generally considered distinct clinical entities. Discovering that both types of meningitis occur simultaneously within the central nervous system (CNS) is rare. The presence of overlapping clinical signs, neuroimaging, and CSF findings complicates the diagnosis. This is particularly true when one pathogen dominates the initial clinical presentation. Thus, a complete microbiological assessment incorporating both the conventional culture methods and molecular techniques is critical in any such case [5].

This case illustrates difficulties in establishing the diagnosis of both forms of meningitis, emphasizes the necessity for maintaining a high index of suspicion in such patients with atypical forms of meningitis, and supports the importance of combining advanced molecular techniques with traditional microbiological approaches for ensuring timely diagnosis and appropriate multidisciplinary management.

## Case presentation

**Figure 1:**
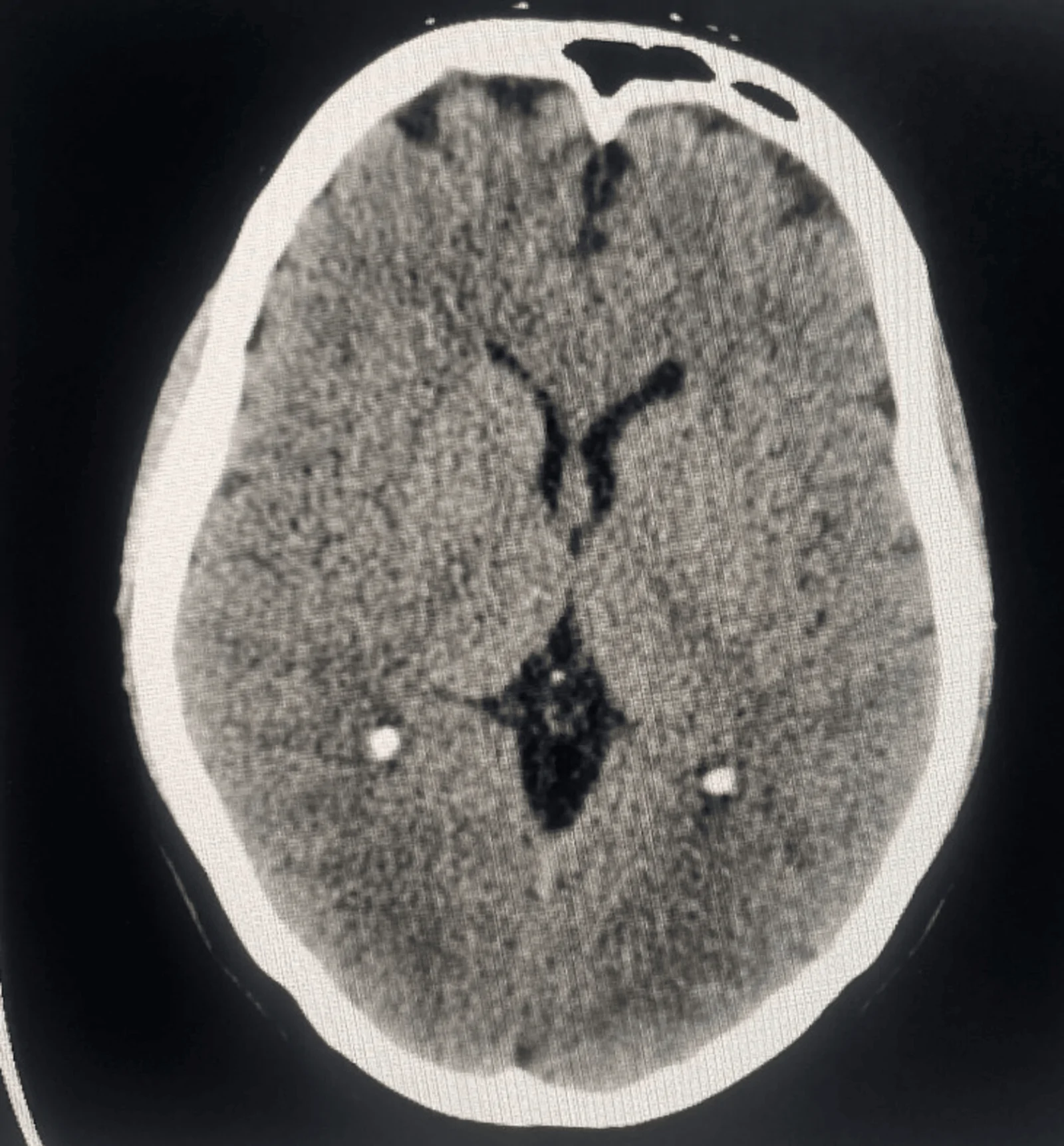
Computed tomography scan of the brain showing no acute intracranial pathology Axial non-contrast computed tomography image demonstrating no evidence of acute intracranial pathology, including intracranial hemorrhage, infarction, mass effect, or hydrocephalus

A 45-year-old male with no prior history of neurological disease presented to the emergency room due to a fever persisting for five days, a severe headache for four days, recurrent vomiting spells of two days' duration, and worsening of sensorium for the last twelve hours corresponding to his arrival at the hospital. His attendants said that the patient lost consciousness following a brief episode of seizures in the morning. Initially, he was assessed at a peripheral health facility, where a computed tomography scan without contrast of the brain showed no evidence of any acute intracranial pathology (Figure 1), and he was subsequently referred as his neurological condition worsened.

Upon arriving at the hospital, the patient had a Glasgow Coma Scale score of 7 (E1V1M5). His heart rate was 130 beats/minute, blood pressure was 170/60 mmHg, while the respiratory rate was 28 breaths/minute, and the oxygen saturation was 98% while receiving 8 liters of supplemental oxygen. Pupils were equal and responsive to stimuli, and neck stiffness was noted. Due to the depressed consciousness and the inability to maintain airway reflexes, emergency endotracheal intubation was carried out through rapid-sequence induction. After the procedure, the patient was transferred to the intensive care unit (ICU) for mechanical ventilation. The initial arterial blood gas (ABG) report showed acute hypercapnic respiratory failure with metabolic acidosis. The laboratory results are depicted in Table 1.

**Table 1:** Laboratory assessments

Laboratory parameter	Result	Units	Reference range
Hemoglobin	13.6	g/dL	12.0-16.0 (female); 13.5-17.5 (male)
Leukocyte count	14,900	cells/mm³	4000-11,000
Neutrophils	95	%	40-75
Platelet count	2.06 × 10⁵	cells/mm³	1.5-4.5 × 10⁵
Creatinine	1.5	mg/dL	0.6-1.3
Urea	18	mg/dL	15-40
Sodium	142.9	mmol/L	135-145
Potassium	3.3	mmol/L	3.5-5.0
C-reactive protein	37.9	mg/L	<5
Prothrombin time	17	seconds	11-15
International normalized ratio	1	-	0.8-1.2

**Figure 2:**
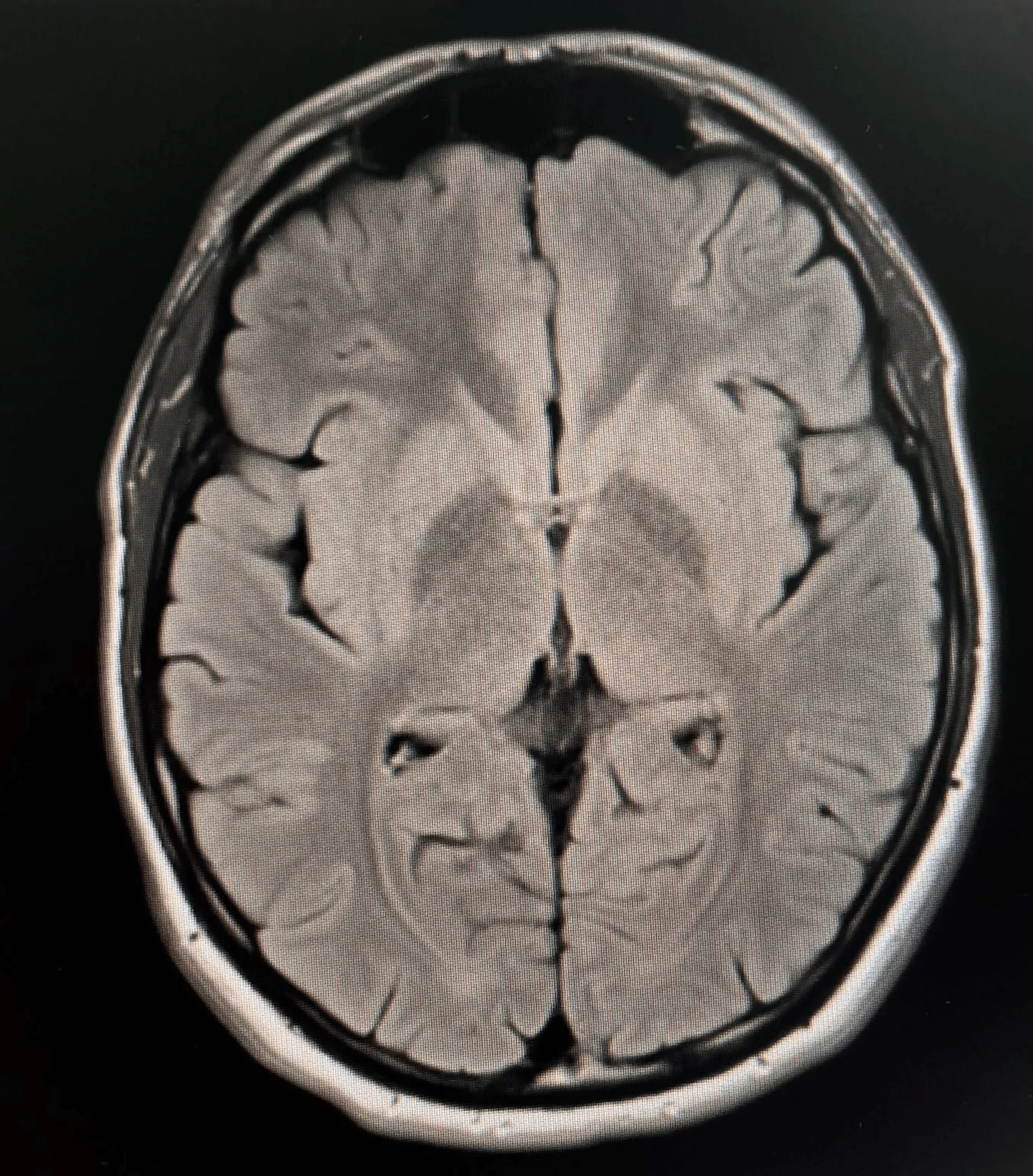
Magnetic resonance imaging demonstrating a normal study Magnetic resonance imaging of the brain demonstrating no evidence of cerebral infarction, intracranial hemorrhage, hydrocephalus, or cerebral abscess

Magnetic resonance imaging of the brain demonstrated no acute intracranial abnormalities (Figure 2). Signs of cerebral infarction, intracranial bleeding, hydrocephalus, and cerebral abscess were absent. After the patient was stabilized, he underwent a lumbar puncture, and slightly turbid CSF was obtained. Analysis of the CSF showed a total leukocyte count of 250 cells/mm³, with 80% lymphocytes and 20% neutrophils, protein concentration of 135.1 mg/dL, glucose level of 94.6 mg/dL, and adenosine deaminase level of 49 U/L. Acid-fast bacilli staining was negative. CB-NAAT performed on the CSF detected *Mycobacterium tuberculosis *(*M. tuberculosis*), while CSF culture isolated *Streptococcus pneumoniae *(*S. pneumoniae*), which was susceptible to ceftriaxone. In view of the acute clinical presentation, inflammatory CSF profile, and discordant microbiological findings, the combined detection of *M. tuberculosis* by CB-NAAT and *S. pneumoniae* by CSF culture supported the diagnosis of concurrent tuberculous and pneumococcal meningitis. The main biochemical, cytological and microbiological indices of the CSF are summarised in Table 2.

**Table 2:** Cerebrospinal fluid analysis Cerebrospinal fluid culture yielded *Streptococcus pneumoniae*, while cartridge-based nucleic acid amplification testing detected *Mycobacterium tuberculosis*

Parameter	Patient value	Reference range
Appearance	Slightly turbid	Clear
Total leukocyte count	250 cells/mm³	0-5 cells/mm³
Differential count	Lymphocytes - 80%, Neutrophils - 20%	Occasional lymphocytes, no significant neutrophils
Protein	135.1 mg/dL	15-45 mg/dL
Glucose	94.6 mg/dL	45-80 mg/dL
Adenosine deaminase	49 U/L	<10 U/L
Acid-fast bacilli stain	Negative	Negative
Cartridge-based nucleic acid amplification test	Detected Mycobacterium tuberculosis	Not detected
Cerebrospinal fluid culture	Growth of Streptococcus pneumoniae	Sterile
Cytology	Inflammatory cells in a proteinaceous background	No abnormal cells

Intravenous ceftriaxone (2 g every 12 hours) and vancomycin (500 mg every eight hours) were started after CSF analysis. Simultaneously, standard four-drug antitubercular therapy comprising isoniazid (300 mg once daily), rifampicin (600 mg once daily), pyrazinamide (1500 mg once daily), and ethambutol (800 mg once daily) was instituted via the enteral route. Additional intensive care management included mechanical ventilation, levetiracetam for seizure prevention, prevention of stress ulcers and deep vein thromboses, enteral nutrition, and the replacement of electrolytes as needed.

**Figure 3:**
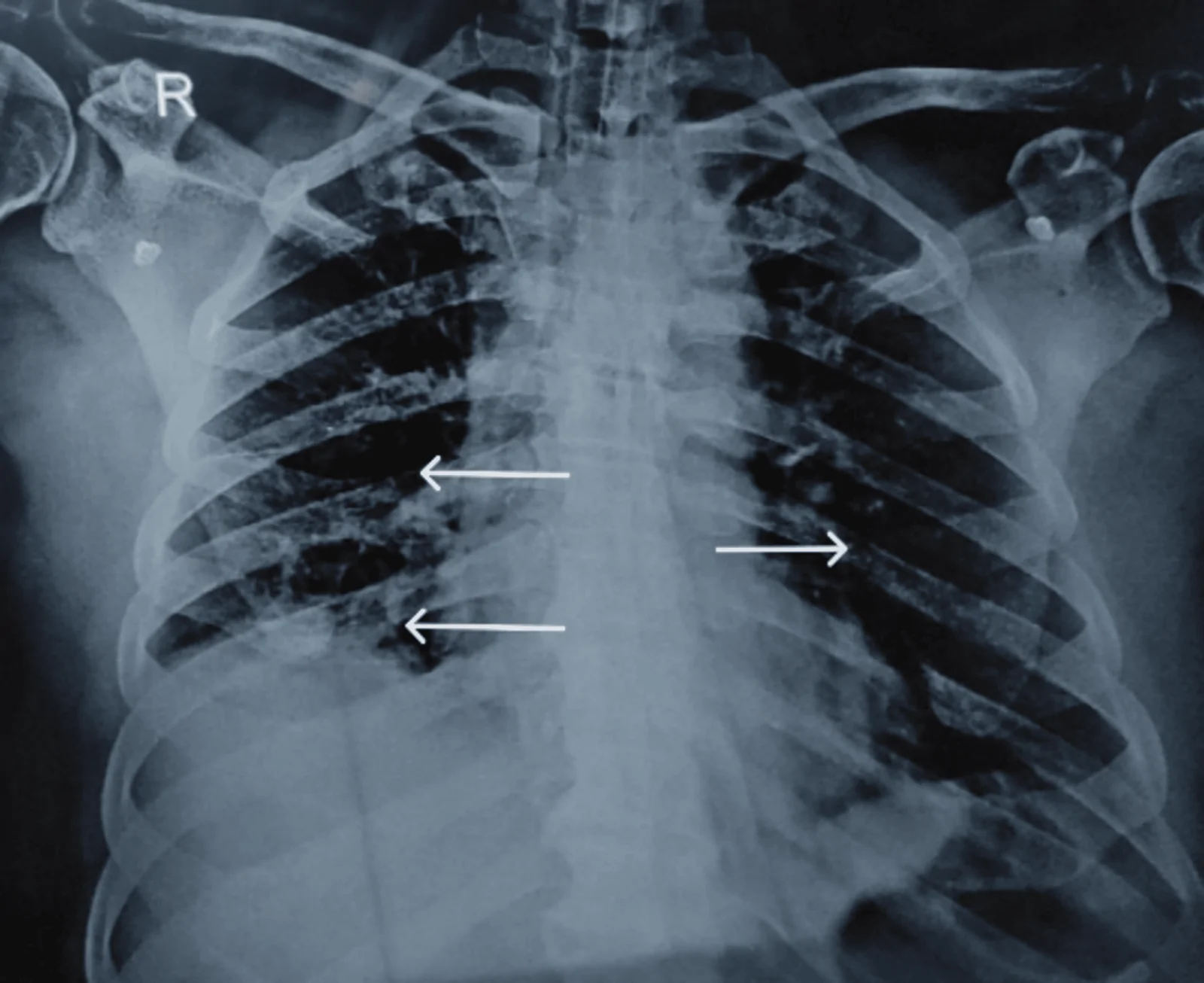
Chest radiograph demonstrating bilateral patchy air-space opacities White arrows indicate bilateral patchy air-space opacities, consistent with multifocal pulmonary infiltrates. Right lower lobe consolidation is also evident

In the course of the ICU admission, the patient experienced persistent fever, large amounts of purulent endotracheal secretions, and an increase in oxygen requirement. Repeated laboratory investigations revealed worsening signs of inflammation, indicated by a leukocyte count of 16,900/mm³ and increased C-reactive protein (CRP) of 77.14 mg/L. Chest radiography demonstrated bilateral patchy air-space opacities with right lower lobe consolidation, suggestive of bronchopneumonia (Figure 3). Endotracheal aspirate culture yielded Klebsiella pneumoniae, which was susceptible to ceftriaxone.

Serial ABG analyses demonstrated resolution of hypercapnia and metabolic acidosis, with normalization of lactate levels and adequate oxygenation. After successful attempts at spontaneous breathing trials, the patient was extubated and maintained adequate oxygen saturation on room air without respiratory distress. After steady neurological improvement, the patient was shifted to the ward on day six, while continuing antitubercular therapy, intravenous antibiotics, and neurological rehabilitation. The patient was discharged from the ward on day 12.

## Discussion

Acute bacterial meningitis and TBM are two different CNS infections, whose pathophysiological processes, CSF profiles, disease course, and treatment regimens differ from each other. While *Streptococcus pneumoniae* remains the most frequent causative agent of community-acquired bacterial meningitis in adults, TBM is the most important form of extrapulmonary tuberculosis and presents very high rates of mortality and long-term neurological consequences for a patient with late diagnosis. Diagnosis becomes complicated when these two kinds of CNS pathologies are observed in one patient, since their clinical picture and laboratory findings can be misleading [1,3,5].

The diagnosis of simultaneous infections of the CNS remains quite difficult, as fever, headache, altered awareness, seizure, and meningeal signs are seen in both acute bacterial meningitis and tuberculosis meningitis. Neuroimaging findings are likewise often nonspecific and do not reliably distinguish between the two entities. The characteristic CSF profiles may overlap when mixed infections occur. While meningitis due to pneumococci typically shows neutrophilic pleocytosis with greatly diminished CSF glucose levels and high protein concentration, TBM usually reveals lymphocytic pleocytosis, increased protein, and decreased glucose. Therefore, unusual CSF results or discrepancies between microbiological findings and clinical picture should raise the suspicion of additional infectious causes and not be attributed only to the rare presentation of one pathogen [1,3,5].

Garg et al. similarly described mixed pneumococcal and TBM, stating that identification of one pathogen should not exclude the search for another in case clinical and lab findings do not match [6]. Similarly, Xiaoli et al. described a case where TBM was diagnosed concurrently with anti-N-methyl-D-aspartate receptor encephalitis [7].

A key factor in the effective management of TBM is accurate diagnosis. The conventional method of acid-fast bacilli smear is limited in sensitivity due to the fact that TBM is paucibacillary. By contrast, CB-NAAT allows for rapid and highly specific detection of Mycobacterium tuberculosis, which in turn enables the prompt initiation of antitubercular treatment. However, it is recommended that molecular tests be used only as a complement to classical microbiological techniques because CSF culture remains crucial for the identification of the causes of pyogenic infections and for measuring the resistance of microbes, while allowing pathogen-directed antibiotic treatment of infections [3,5].

Mahashabde et al. demonstrated that a comprehensive microbiological examination is necessary in order to diagnose concurrent TBM and cryptococcal meningitis and that relying on one test is likely to miss co-infection [8]. Likewise, Bi et al. showed that an integrated approach combining classical microbiological techniques with sophisticated molecular diagnostics made it possible to correctly diagnose concurrent tuberculous, cryptococcal, and streptococcal meningitis [9].

Successful management of dual CNS infections requires prompt initiation of pathogen-directed antimicrobial therapy together with comprehensive neurocritical care. The use of adjunctive corticosteroids remains an important concern of TBM treatment as they lessen the injuries caused by inflammation, while the timely administration of required antibiotics is necessary for successful management of pneumococcal meningitis. Patients requiring prolonged intensive care are also vulnerable to hospital-acquired infections, especially ventilator-associated pneumonia, which warrants ongoing microbiological monitoring, antimicrobial stewardship, and a multidisciplinary approach during hospitalization. Early detection of secondary infections alongside timely modification of antibiotic therapy plays an equally paramount role in improving treatment outcomes [1-3].

This case underscores the importance of maintaining a high index of suspicion for concurrent CNS infections when the clinical course, neuroimaging findings, or CSF profile cannot be fully explained by a single diagnosis.

## Conclusions

Simultaneous occurrence of TBM and pneumococcal meningitis is a rare but possibly fatal clinical condition that presents significant diagnostic and therapeutic dilemmas due to similar clinical presentation and CSF characteristics. This case reinforces that identification of one pathogen should not preclude investigation for additional etiologies when the clinical presentation or CSF findings remain atypical. Comprehensive CSF evaluation using both molecular and conventional microbiological techniques is essential for timely recognition of dual infections and initiation of appropriate pathogen-directed therapy.
